# Performance Analysis of Turbo Codes, LDPC Codes, and Polar Codes over an AWGN Channel in the Presence of Inter Symbol Interference

**DOI:** 10.3390/s23041942

**Published:** 2023-02-09

**Authors:** Adriana-Maria Cuc, Florin Lucian Morgoș, Cristian Grava

**Affiliations:** Department of Electronics and Telecommunications, University of Oradea, 410087 Oradea, Romania

**Keywords:** equalization, Zero Forcing, minimum mean square error, least square

## Abstract

This paper discusses the results of simulations relating to the performances of turbo codes, low density parity check (LDPC) codes, and polar codes over an additive white Gaussian noise (AWGN) channel in the presence of inter symbol interference, denoting the disturbances that altered the original signal. To eliminate the negative effects of inter symbol interference (ISI), an equalizer was used at the level of the receiver. Practically, two types of equalizers were used: zero forcing (ZF) and minimum mean square error (MMSE), considering the case of perfect channel estimation and the case of estimation using the least square algorithm. The performance measure used was the modification of the bit error rate compared to a given signal to noise ratio; in this sense, the MMSE equalizer offered a higher performance than the ZF equalizer. The aspect of channel equalization considered here is not novel, but there have been very few works that dealt with equalization in the context of the use of turbo codes, especially LDPC codes and polar codes for channel coding. In this respect, this research can be considered a contribution to the field of digital communications.

## 1. Introduction

The theory of information transmission has experienced huge growth since the work of Shannon (1948) demonstrated the theoretical possibility of reliable transmission [1]. 

Two main classes of technique make it possible to ensure the reliability of a transmission: channel coding, which aims to code the transmitted message in such a way that the receiver can correct most transmission errors; and equalization, the objective of which is to make the best use of the bandwidth of the medium (transmission channel) [2,3]. 

In practical communication systems, transmitted signals are subject to various distortions, such as noise, inter-symbol interference (ISI) due to multipath propagation, and the nonlinear effects of amplifiers. All these factors alter the original signal, so it is essential to restore the channel’s input by observing its output. The process of restoring the original signal consists of the application of a channel equalizer [4,5]. 

This is where the techniques of equalization and channel estimation come in, the purpose of which is to determine the disturbances of the channel to eliminate them or at least limit their impact on the transmitted signal. These systems are sized to optimize certain transmission quality criteria, thanks to very powerful mathematical tools and sophisticated algorithms.

The role of the equalizer filter is to use the observations of the channel to restore the transmitted signal and reduce the distortions caused by the channel as much as possible [6,7,8,9,10,11].

The problem is to estimate the values of the coefficients of the equalizer filter, to allow restoring the transmitted signal with a low probability of possible error. 

An optimization of the coefficients of the equalizer filter that minimizes in the most precise manner the probability of the appearance of these errors is strongly desired. Several criteria for optimizing these coefficients are recommended; the most used being that of minimizing the mean square error (MMSE). Although an MMSE equalizer provides a good performance, a simple approach to designing a zero forcing equalizer at the level of the receiver was considered in [12].

Optimization theory also offers various algorithms; examples include gradient algorithms, the Viterbi algorithm, etc. [10,13].

The choice of algorithm is essentially linked to the choice of the criterion. The Viterbi algorithm, for example, is applied to a maximum likelihood criterion, while the gradient algorithms are related to non-linear criteria, such as the mean square error (MSE) [14,15]. The choice of structure is determined by both the criterion and the optimization algorithm. Two fundamental structures are presented in the literature: the transverse structure, and the lattice structure. These structures are designed using finite impulse response filters (FIR) or infinite impulse response (IIR) [15]. Structures of the non-linear type are filter-based but have a non-linear behavior, such as architectures with decision feedback [10,15].

These equalizers have the main function of inverting the response of the transmission channel, so that the “channel–equalizer” couple can be considered an ideal channel. The equalization operation can cause, depending on the nature of the equalizer used, more or less significant increases in the noise present at its input [10]. 

This research investigates the situation when error correcting codes, such as turbo codes, LDPC codes, and polar codes are applied over the AWGN channels that present ISI. More precisely, it was proposed to research the equalization and the estimation of an AWGN channel in the context of the use of the three most efficient codes; and indeed the turbo codes, LDPC codes, and polar codes proposed in [16,17,18] make it possible to achieve performances quite close to the Shannon limit, even over the AWGN channels. Some aspects related to coding and decoding the fields of use of turbo codes, LDPC codes, and polar codes were treated in [19,20]. In a few words, two recursive systematic convolution encoders separated by an interleaver are used to build a turbo encoder. Polar codes are constructed based on the concept of channel polarization. In the case of decoding, the turbo decoder is based on the BCJR (Bahl, Cocke, Jelinek, and Raviv) or MAP algorithms [16]. On the other hand, the decoding of polar codes is based on the successive cancellation algorithm [18]. Regarding LDPC encoding, both the base matrix and expand factor were utilized in order to exemplify the parity check matrix H [17]. The encoding of LDPC in the 5G standard implied the usage of a base matrix of 42 × 52 size (base graph 2). In preparation for LDPC, codes decoding the algorithm of sum product (or message passing) was applied.

The simple enhancements made in this paper to 5G LDPC codes, polar codes, and 3G and 4G turbo codes using equalization techniques could also be considered, for example, to improve performance in the field of digital communications.

The landmark codes such as turbo, LDPC, and polar are widely used in modern communication system standards. Starting from the general results obtained in Section 5 of this paper, further results could be obtained using specific standards such as 3GPP-LTE, WiMAX, and WiFi, concerning turbo codes, or 5G machine type communication applications, such as ultra-reliable low-latency communications (URLLC) and massive machine-type communication (mMTC) concerning the LDPC and polar codes [19,20]. Several decoding and equalization receiver schemes designed for different standards have been proposed in the literature [21,22,23].

Depicting the performance of AWGN channels in the presence of ISI, the paper focused on only the most advanced forward error correction schemes, such as turbo codes, LDPC codes, and polar codes, due to their great effect on the existing communications standards (long term evolution (LTE) and 5G standards) and their high efficiency, since they can perform near the channel capacity [24]; this analysis also applies to the other codes, such as RS codes (Reed–Solomon), Raptor codes, and so on.

The main purposes of this paper are summarized as follows:

- This paper presents a novel LDPC least square (LS) channel estimator, using the LLR (log-likelihood) fed back from the LDPC decoder to iteratively improve the channel estimation.
- In addition, this paper presents a novel polar least square (LS) channel estimator, achieved based on the sequence obtained at the output of the polar decoder to iteratively improve the channel estimation.
- In the working SNR range, the iterative LS estimation of the channel response to the impulse offers substantial improvements regarding the performance of all three types of codes (LDPC codes, polar codes, and turbo codes) compared to estimation only once, and the LDPC codes seemed to give the best results. Additionally, computer simulation results are presented to confirm this assertion.

It should be noted that there are works, such as those previously mentioned, that individually evaluated the performance of turbo codes, LDPC codes, or polar codes using specific standards, but this paper is the first to highlight the performance of the three codes simultaneously using the same AWGN channel model and applying different equalization techniques. It BPSK (binary phase-shift keying) modulation was assumed in this paper, but the analysis could be applied to other modulation methods as well. Thus, in future, these results will help in choosing or implementing different types of codecs, depending on the application.

For the moment, this integrative paper can be considered a good starting point for researchers who are focused on increasing the accuracy of the application of signal equalization techniques at the reception side.

## 2. Related Work

In practical communication systems, transmitted signals are subject to various distortions, such as noise, inter symbol interference due to multipath propagation, and the nonlinear effects of amplifiers. All these factors alter the original signal, so it is essential to restore the input of the channel by observing its output [25,26,27].

The process of restoring the original signal uses a filter such as a finite impulse response filter as a channel equalizer [28,29]. The role of the equalizer filter is to use the observations/estimations of the channel to restore the transmitted signal and reduce the distortions caused by the channel as much as possible [7,10,15,30].

The main issue consists of estimating the values of the coefficients of the equalizer filter, to be able to restore the transmitted signal with a low probability of error. To achieve this fact, in this work, the channel model was first estimated, and using this estimate, adjustment of the equalizer filter coefficients was then optimized.

The optimization of these coefficients that best minimizes the probability of the appearance of errors is strongly desired. Several optimization criteria are proposed in this paper, such as ZF and MMSE.

Channel equalization being achieved before the decoding process is considered a significant and advanced issue in wireless communication [30].

In this respect, in [23], the authors realized a BER vs. SNR performance comparison of a turbo MMSE equalizer for uplink narrowband Internet of Things systems using BPSK and QPSK (quadrature phase shift keying) with a different number of iterations. The simulations were performed in Matlab. The analysis proved that the performance of QPSK modulation was comparable with the performance of BPSK modulation.

In [31], the authors proposed expectation propagation (EP) as a turbo equalizer, to offer a reliable estimate that, in comparison with linear MMSE equalization, revealed a better performance. This newly proposed equalizer presented gains in the range of 1.5–5 dB in contrast to linear minimum mean square error equalization.

A new method was presented in [32] for executing an adaptive DFE (decision feedback equalizer). Whereby, both least square and recursive least square algorithms were employed to predict the channel and adjust the equalizer vector, jointly. The performance of the proposed algorithm was close to the performance of maximum likelihood equalization.

An investigation regarding the usage of decision feedback with a turbo equalizer to improve the limits of linear equalizers was undertaken in [33]. The turbo DFE using hard feedback with symbol-wise adaptive filters performed poorly at low spectral efficiency, whereas the turbo DFE using soft posterior feedback with symbol-wise invariant filters was outperformed by time-varying linear equalizers-interference cancellers.

A global iteration method using feedback among LDPC and a LT decoder in the raptor code was applied in [34] to improve the BER performance. The authors proposed a joint equalization and raptor decoding algorithm, which could use the updated information from raptor decoders.

The performance of LDPC codes and polar codes with channel equalization were evaluated in [35] using free-space optical transmission experiments, demonstrating that the BLER (block error rate) of polar codes was better than that of LDPC codes.

Polar codes with adaptive channel equalization were adopted in [36], to decrease the feedback information. In this respect, a hybrid automatic repeat request mechanism was applied to moderate the effect of estimation errors. The results showed that the proposed scheme outperformed the turbo equalization in terms of BER.

## 3. Inter Symbol Interference

Inter symbol interference (ISI) generally results from limited bandwidth being allocated to a channel, the presence of nonlinearities due to power amplifiers, and distortions due to multipath propagation. Indeed when the signal enters the channel, it suffers disturbances such as the addition of noise or its reflection by obstacles. This is equivalent to transmitting the signal through several separate channels, each having a different attenuation and delay [37]. 

**Figure 1:**
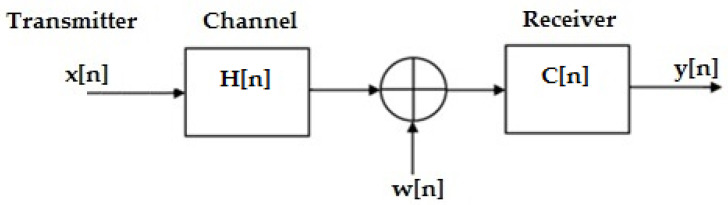
The digital transmission chain [10].

Practically, the recipient receives the direct signal and also the contribution of the attenuated and delayed signals, each having followed a different path; this refers to inter symbol interference. The modeling of the digital transmission chain is represented in Figure 1:

In Figure 1 the useful signal x[n], is transmitted over a channel with ISI, H(n), to which an additive white noise w(n) has been added. On the receiver side, the received signal is sent over an equalizer C(n) to minimize the effect of ISI.

The signal y(n), corresponding to the channel output, can be represented as a discrete-time equation, such as [38]

(1) $$y(n)=\underset{\mathrm{ISI}}{\underbrace{\sum_{k=0}^{\infty }h(k)x(n-k)}}+w(n),$$

Regarding Equation (1), the convolution is represented by the weight function h(k), which moves over the input signal x(n), resulting in a sum of the input samples weighted by the filter coefficients, and w(n) represents the additive noise.

If the h(0) term of the sum is written individually, the equivalent form of (1) can be written as

(2) $$y(n)=h(0)x(n)+\underset{\mathrm{ISI}}{\underbrace{\sum_{\begin{array}{l} k=1\\ k\neq n \end{array}}^{\infty }h(k)x(n-k)}}+w(n),$$

In Equation (2), the first term x(n) represents the useful signal at time n, the second term is the inter-symbol interference (ISI) representing the contribution of neighboring symbols, and the last term denotes the additive noise.

Consequently, it can be concluded that if a response h(k) is the following:

(3) $$h(k)=\{\begin{array}{cc} 1; & k=0\\ 0; & k\neq 0 \end{array},$$

then the received signal y(n) at each sampling instant is influenced absolutely by the transmitted symbol, therefore eliminating all ISI. Thus, this is the time domain requirement for a channel without ISI.

The signal transmitted through the channel suffers various distortions (such as inter symbol interference). To resolve this issue, an equalizer is employed, for instance, a filter positioned at the output of the transmission channel, to fix/recover the transmitted signal [39].

Different circumstances and constraints in the environment of communications affect the signal transmitted and these also cause the phenomenon of ISI, which consists of a partial overlapping of specific adjacent symbols, thus causing damaging effects to the signal. To limit the effect of the ISI, an equalizer cans be situated downstream of the transmission chain, right before the decision block.

**Figure 2:**
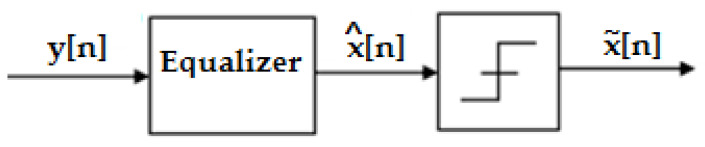
The decision block is preceded by an equalizer.

Practically, the general objective is to apply an equalizer filter C(n) to the samples y(n), to restore the equivalent channel H(n). Here, $\hat{x}(n)$ represents the output corresponding to the equalizer, and $\tilde{x}(n)$ represents the output of the decision block corresponding to the estimate of the input signal x(n). Figure 2 illustrates this construction.

**Figure 3:**
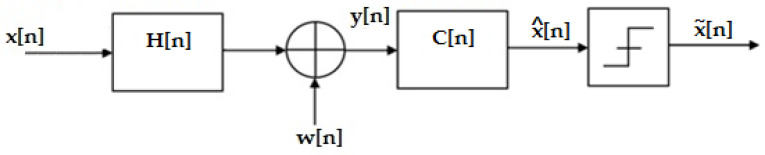
Discrete channel model [10].

Subsequently, the equivalent discrete channel model will be considered, of which the configuration is presented in Figure 3.

The equivalent discrete channel H(n) is defined by the L nonzero coefficients h(k).

The linear time-invariant system is characterized by the associated weight function h(n), which represents the impulse response of the system, such as the output corresponding to a Dirac impulse applied to the input [40].

The operation performed by a filter block is effectively a convolution operation. The convolution is represented by the weight function h(n), which overlays the input signal, resulting in the sum of the input samples being weighted by the filter coefficients. The sum is the response of the filter at a certain moment. 

Therefore, a noisy output of the equivalent discrete channel y(n) is given by:

(4) $$y(n)=\sum_{k=0}^{L-1}h(k)x(n-k)+w(n),$$

The C(n) equalizer is defined by the $C_{k}$ coefficients, and its output $\hat{x}(n)$ is the sum of the received signal samples weighted by the equalizer coefficients. Therefore, the equalizer output $\hat{x}(n)$ is given by:

(5) $$\hat{x}(n)=\sum_{k=0}^{L-1}C_{k}y(n-k),$$

It is useful to adopt matrix notation for the equalizer output, such as:

(6) $$\hat{x}(n)=\sum_{k=0}^{L-1}C_{k}y(n-k)=C^{T}y(n)=y^{T}(n)C,$$

where $C={\left[\begin{array}{cccc} C_{0} & C_{1} & \ldots & C_{L-1} \end{array}\right]}^{T}$ is a vector of length L containing the coefficients of the equalizer, and $y(n)=[\begin{array}{cccc} y(n) & y(n-1) & \ldots & y(n-L+1) \end{array}]$ is the vector of the most recent input data.

The equalization function makes it possible to find from the received sequence y(n), which is affected by ISI, the transmitted sequence x(n) [41].

## 4. Design of the Equalizers

An equalizer is a filter placed at the receiver layer in the transmission chain, whose main task is to adjust, as much as possible, the signal at its output to match that which had been transmitted. 

This is possible by applying a model that controls the relationship between the input and the output of the filter. 

There are two architectures of equalizers: linear and non-linear [15].

### 4.1. Linear Equalizer

**Figure 4:**
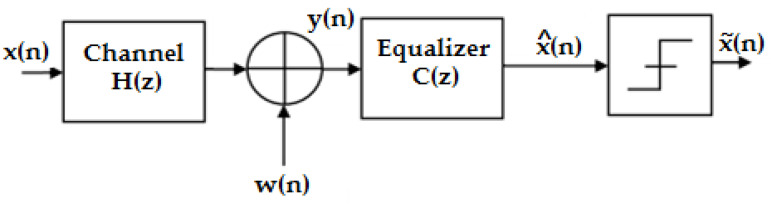
Diagram of a linear equalizer [15].

A linear equalizer is a finite impulse response (FIR) filter with adjustable coefficients followed by a threshold decision circuit. Figure 4 illustrates the principle of the linear equalizer, whose coefficients can be optimized using one of the equalization rules that will be presented in this paper. 

H(z) and C(z) represent the z-transforms regarding the channel filter and the equalizer filter, respectively.

The output of the equalizer is related to its input using the following equation:

(7) $$\hat{x}(n)=\sum_{k=-\infty }^{+\infty }c_{k}y(n-k),$$

### 4.2. Nonlinear Equalizer

Although nonlinear equalizers are not the subject of this paper, it can be mentioned that it is considered another type of equalizer architecture. An example of a nonlinear equalizer is the decision feedback equalizer (DFE). This consists of a transverse filter and a recursive one [42]. 

**Figure 5:**
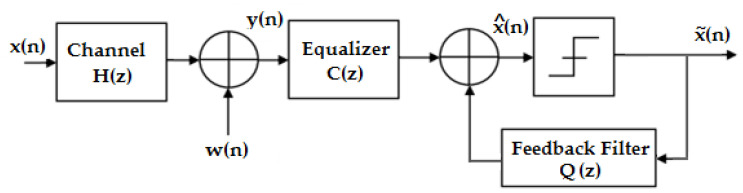
Diagram of a nonlinear equalizer [15].

The input of this filter is represented by the symbols determined previously, and which are used to eliminate the ISI from the current estimate. A block diagram of the decision feedback equalizer is given in Figure 5.

In this structure, C(z) mainly serves to minimize the ISI on the current symbol caused by the future symbols, while the recursive part Q(z) synthesizes the ISI caused by the symbols passed and subtracts it from the signal before the decision.

The DFE equalizer output is expressed as

(8) $$\hat{x}(n)=\sum_{k=-\infty }^{0}c_{k}y(n-k)+\sum_{k=1}^{+\infty }q_{k}\tilde{x}(n-k),$$

There are two rules for determining the optimal settings for these equalizers: the forcing criterion to zero, and the minimum mean square error [15].

### 4.3. Zero Forcing Equalizer

The term zero forcing (ZF) signifies reducing ISI to zero. The filtering operation usually represents the transmission of a signal x(n) through a time invariant linear system, whose weight function h(n) is known.

**Figure 6:**
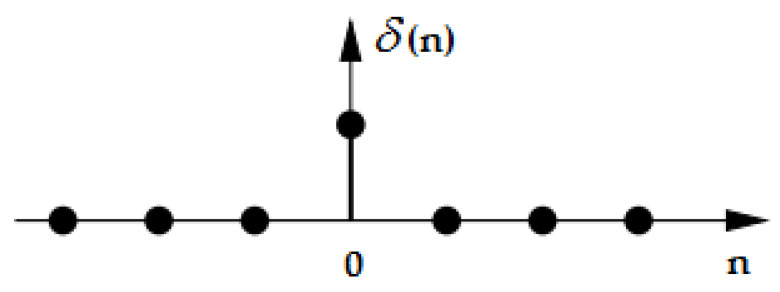
Unit impulse or unit sample.

When applying ZF, the purpose is to determine the equalizer impulse response c(n), in such a way that using the convolution operation with the weight function of the system h(n) can obtain the unit impulse (which takes the value 1 for index 0 and 0 otherwise between −∞ and +∞, as Figure 6 depicts), thus canceling the interference caused by the channel [43], according to

(9) h(n)∗c(n)=δ(n),

Equation (9) can be transcribed as a group of linear equations, obtaining

(10) $$q_{m}=\sum_{n}c_{n}h_{m-n}=\{\begin{array}{cc} 1; & m=0\\ 0; & m=\pm 1,\pm 2,\ldots ,\pm N \end{array},$$

This formula can be written as a matrix equation, respectively

(11) Tc=q,

where T is a Toeplitz matrix whose elements match the following statement:

(12) $$T_{\mathrm{ij}}=t_{i-j},$$

otherwise, it can be written as

(13) $$T=\left[\begin{array}{ccccc} t_{0} & t_{-1} & t_{-2} & \ldots & t_{1-n}\\ t_{1} & t_{0} & t_{-1} & \ldots & t_{2-n}\\ t_{2} & t_{1} & t_{0} & \ldots & t_{3-n}\\ \ldots & \ldots & \ldots & \ldots & \ldots \\ t_{n-1} & t_{n-2} & t_{n-3} & \ldots & t_{0} \end{array}\right],$$

Thus, the coefficients of the equalizer are determined by reversing the square matrix T, according to:

(14) $$c={\mathrm{qT}}^{-1},$$

It can be noticed that noise is neglected in the development of the ZF criterion. In practice, however, noise is always present, and although the ISI terms are eliminated, there is the possibility that the equalizer amplifies the effect of the noise and therefore degrades the performance [44].

It is also worth noting that the channel is assumed to be perfectly known, consequently, the errors in the estimation of the channel impulse response will then affect the equalizer coefficients and will lead to a degradation of performance. 

The zero-force principle (ZF) cancels the inter-symbol interference but does not achieve good performance, because it does not take into account the noise through the channel.

### 4.4. Minimum Mean Square Error Equalizer

Unlike the ZF, a minimum mean square error (MMSE) equalizer integrates the effect of noise into the criterion it attempts to optimize. Its purpose is to minimize the mean square error (MSE) between the transmitted sequence x(n) and its estimate at the output of the equalizer $\hat{x}(n)$.

Therefore, using the MSE criterion to determine the optimum coefficients of the equalizer is based on the minimization of the following function [15]:

(15) $$J=E\left\{{\left|e(n)\right|}^{2}\right\},$$

where the error is given by

(16) $$e(n)=x(n)-\hat{x}(n)=x(n)-C^{T}y(n),$$

Therefore, the cost function can be written as

(17) $$J(C)=J=E\left\{e^{2}\left(n\right)\right\}=E\left\{x^{2}\left(n\right)\right\}-2E\left\{{x(n)y}^{T}\left(n\right)\right\}C+C^{T}E\left\{{y(n)y}^{T}\left(n\right)\right\}C,$$

By replacing the terms in Equation (17) as follows: $E\left\{{y(n)y}^{T}\left(n\right)\right\}=R$; E{y(n)x(n)}=P; and $E\left({\left|x_{k}\right|}^{2}\right)=\sigma_{x}^{2}$; where R represents the covariance matrix of the received signal, P is the correlation vector between the received signal and the transmitted one and $\sigma_{x}^{2}$ is the variance of the transmitted signal, and Equation (17) can be written as

(18) $$J(C)=\sigma_{x}^{2}-{2P}^{T}C+C^{T}\mathrm{RC},$$

J(C) is a quadratic function of the parameters $\left\{C_{0}{,C}_{1},\ldots {,C}_{L-1}\right\}$. Function J(C) has a unique minimum, obtained by taking the gradient equal to zero, such as

(19) $$\partial J(C)/\partial C=-P+C^{T}R=0,$$

from where it results in

(20) $$C_{\mathrm{opt}}=R^{-1}P,$$

Equations (19) and (20) are known as the Wiener–Hopf equations.

At the optimum, it can be written that

(21) $${J(C}_{\mathrm{opt}})=\sigma_{x}^{2}-{2P}^{T}C_{\mathrm{opt}}+C_{\mathrm{opt}}^{T}{\mathrm{RC}}_{\mathrm{opt}}=\sigma_{x}^{2}-C_{\mathrm{opt}}^{T}{\mathrm{RC}}_{\mathrm{opt}}=\sigma_{x}^{2}-P^{T}R^{-1}P=\sigma_{x}^{2}-\sigma_{\mathrm{xopt}}^{2}$$

where $x_{\mathrm{opt}}\left(n\right)=C_{\mathrm{opt}}^{T}y(n)$ is the optimal filtered signal and $\sigma_{\mathrm{xopt}}^{2}=E\left\{x_{\mathrm{opt}}^{2}\left(x\right)\right\}$ is the variance of this signal.

Consequently, the relation (21) asserts that for the optimal filter, the MSE is the difference between the variance of the transmitted signal and the variance of the estimated signal produced by the filter.

Thus, the value of MMSE for the optimal Wiener filter is according to

(22) $$J_{\min}={J(C}_{\mathrm{opt}}),$$

### 4.5. Least Square Method for Channel Estimation

The vast majority of the existing methods for estimating the channel with ISI are based on the least square (LS) method or the minimum mean square error (MMSE).

The LS estimation method consists of finding the ĥ that minimizes the squared error by applying the following equation [45]:

(23) $$\hat{h}=\underset{h}{\arg\;\min}{\left\|\tilde{s}-\mathrm{Sh}\right\|}^{2},$$

Supposing that the noise is white and Gaussian, the solution to Equation (23) can be represented as follows:

(24) $${\hat{h}}_{\mathrm{LS}}={{(S}^{T}S)}^{-1}S^{T}\tilde{s},$$

where $S=\left[\begin{array}{cccc} s_{L} & \ldots & s_{1} & s_{0}\\ s_{L+1} & \ldots & s_{2} & s_{1}\\ \vdots & \vdots & \vdots & \vdots \\ s_{L+N-1} & \ldots & s_{N} & s_{N-1} \end{array}\right]$ is the Toeplitz matrix that corresponds to the transmitted training sequence $s=[\begin{array}{cccc} s_{0} & s_{1} & \ldots & s_{N+L-1} \end{array}]$, and $S^{T}$ represents the transposition of the matrix S.

**Figure 7:**
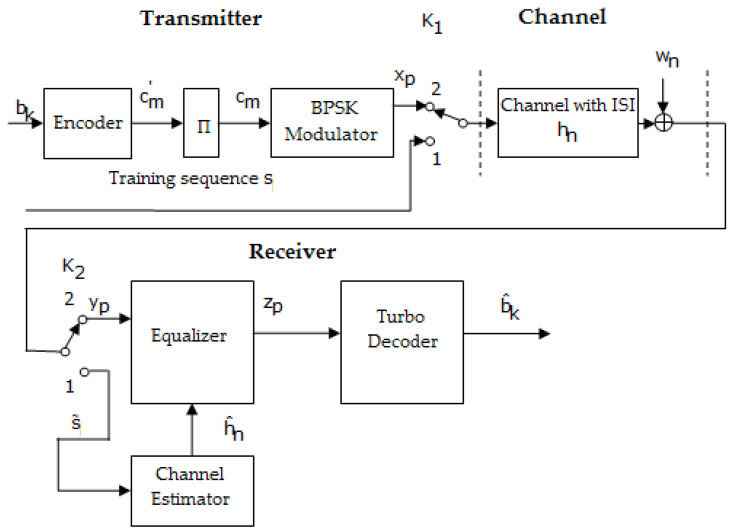
Block diagram of the transmission–reception system that performs the estimation and the equalization of the channel at the reception, in the case of applying turbo codes [46].

Figure 7 depicts a block diagram of the transmission–reception system, wherein the estimation and the equalization of the channel are performed on the reception side, in the case of using turbo codes.

Related to Figure 7, Π represents the inter-leaver block; $c_{m}^{\prime }$ represents the parity bits and the bits of information; $c_{m}$ is the parity bits, the bits of information, and the parity bits of the interleaved sequence; $x_{p}$ is the sequence obtained after BPSK modulation; $y_{p}$ denotes the received sequence; $z_{p}$ denotes the equalized sequence; ${\hat{b}}_{k}$ represents the estimated information bits; and $\tilde{s}$ is the received training sequence.

**Figure 8:**
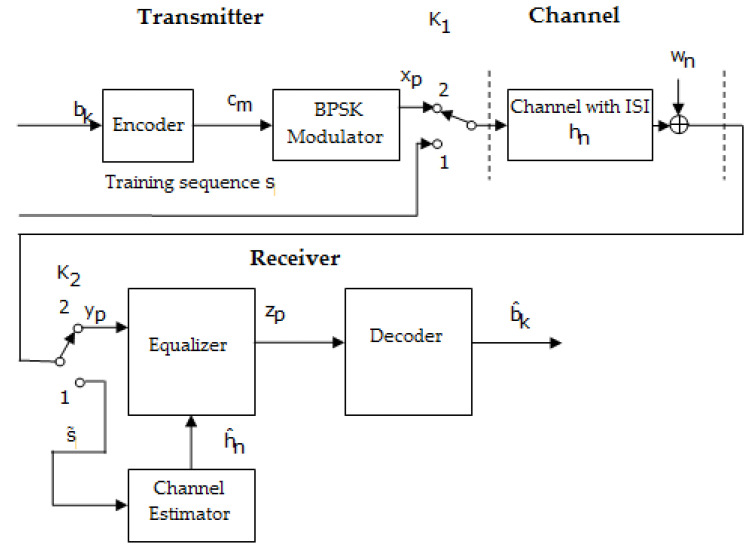
Block diagram of a transmission–reception system that performs the estimation and the equalization of the channel at the reception, in the case of applying LDPC codes and polar codes [46].

On the other hand, in the case of adopting other encoding and decoding techniques regarding LDPC codes and polar codes, the transmission–reception system, shown in Figure 8, is different from the previous one, because of the lack of an inter-leaver at the transmitter.

The two block diagrams shown in Figure 7 and Figure 8 works in the same manner. Thus, when the switches are in position 1, a training sequence s is transmitted; this is known as the channel estimator.

Based on the received sequence and using an estimation algorithms such as LS, the estimated value of h($\hat{h}$) can be determined. When the switches are in position 2, data are transmitted.

**Figure 9:**
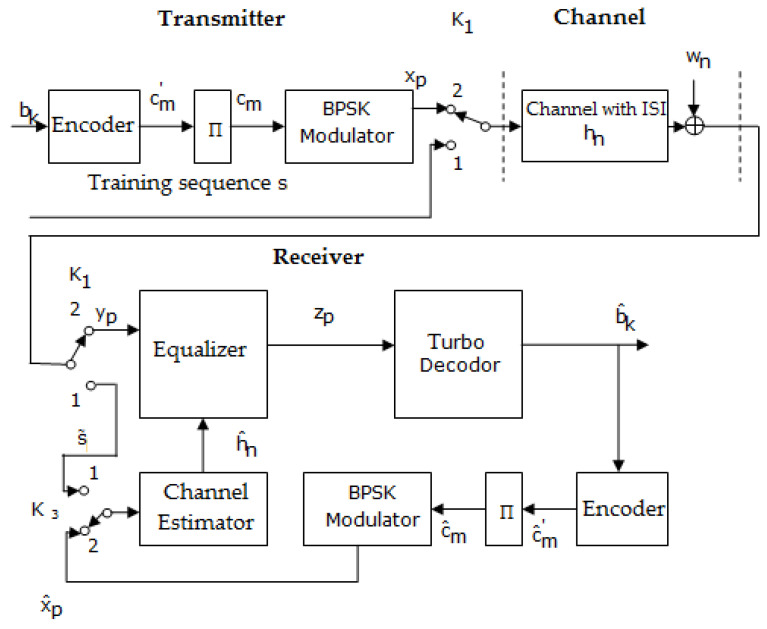
Block diagram of a transmission–reception system that performs the iterative estimation and the equalization of the channel at the reception, in the case of applying turbo codes [46].

An improvement of the turbo channel estimation, and implicitly of the turbo decoding, can be obtained using the block diagram depicted in Figure 9. It depicts the iterative estimation in the case of applying turbo codes.

**Figure 10:**
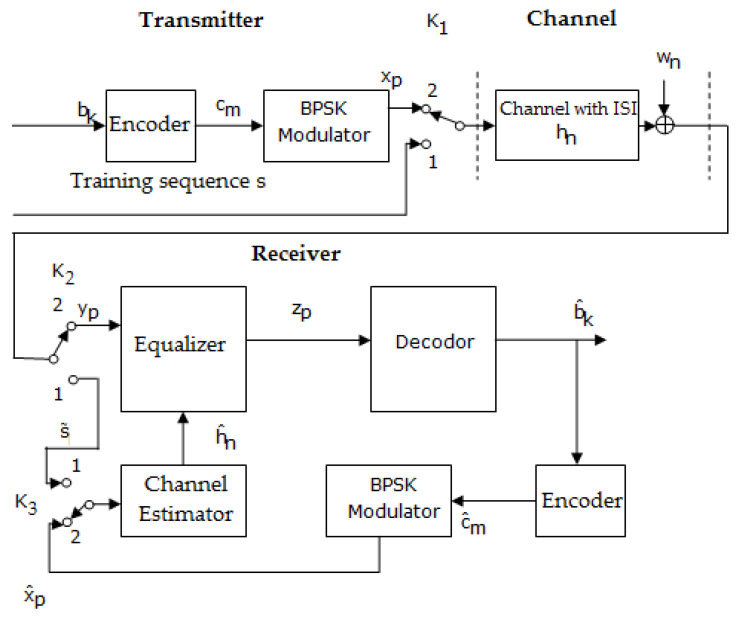
Block diagram of a transmission–reception system that performs the iterative estimation and the equalization of the channel at the reception, in the case of applying LDPC codes and polar codes [46].

In addition, in Figure 10 is presented a block diagram of performing the iterative estimation and the equalization of the channel at the reception, in case of applying LDPC codes and polar codes. 

Practically, comparing the schemes depicted in Figure 7 and Figure 8 with the ones presented in Figure 9 and Figure 10 shows that the difference resides in the fact that the channel estimation is done iteratively in the latter Figures, in contrast to the first ones, where the channel estimation is performed once.

For example, in Figure 9, after a certain number of iterations imposed by the turbo decoder, the sequence estimated ${\hat{b}}_{k}$ at its output is encoded and interleaved, and then BPSK (binary phase-shift keying modulation) modulated and brought to the channel estimator. Then, the estimator channel based on the received sequence $y_{p}$ and the transmitted estimated sequence ${\hat{x}}_{p}$ recalculates the value of ${\hat{h}}_{n}$ using the LS estimation algorithm, according to

(25) $${\hat{h}}_{\mathrm{LS}}={{(\hat{X}}^{T}\hat{X})}^{-1}{\hat{X}}^{T}y,$$

where $\hat{X}$ is the Toeplitz matrix corresponding to the sequence $\hat{x}[p]$, which enters the LS estimator, and ${\hat{X}}^{T}$ represents the transposition of the matrix $\hat{X}$.

If it is assumed that

(26) $$\hat{x}[p]=\left[\hat{x}\left[1\right],\;\hat{x}\left[2\right],\ldots ,\;\hat{x}\left[p\right]\right],$$

and the received signal is

(27) y[p]=[y[1],y[2],…,y[p]],

and the estimated value of h is

(28) $${\hat{h}}_{\mathrm{LS}}={\left[\hat{h}\left[1\right],\;\hat{h}\left[2\right],\ldots ,\;\hat{h}\left[n\right]\right]}^{T},$$

then, the matrix $\hat{X}$ looks like this

(29) $$\hat{X}=\left[\begin{array}{cccc} \hat{x}\left[1\right] & 0 & \ldots & 0\\ \hat{x}\left[2\right] & \hat{x}\left[1\right] & \ldots & 0\\ \vdots & \vdots & \vdots & \vdots \\ \hat{x}\left[p\right] & \hat{x}\left[p-1\right] & \ldots & \hat{x}\left[p-n+1\right] \end{array}\right],$$

## 5. Simulation Results and Discussion

To validate the theoretical models described in the previous chapters, their capabilities were examined for digital transmission, applying binary phase-shift keying modulation (BPSK).

The simulations were performed using MATLAB software.

In this part, the results obtained for the comparison of the turbo codes, LDPC codes, and polar codes in the presence of an AWGN channel with ISI are presented. The code rate $R=\frac{1}{3}$ was considered for the performance comparison of turbo codes, LDPC codes, and polar codes.

Practically, the performance of all three of the previously mentioned codes was compared from the perspective of the proposed approaches, such as the perfect channel estimation, with those of the estimation with the LS (least square) algorithm, in terms of BER vs. SNR (bit error rate vs. signal to noise ration). 

Concerning the estimation with the LS algorithm, both iterative estimation and estimation only once were considered for the comparison of performance of the turbo codes, LDPC codes, and polar codes, in terms of BER vs. SNR.

First, the case of perfect channel estimation was analyzed, meaning that the impulse response of the channel h is well known by both transmitter and receiver.

Therefore, the model of the equivalent discrete channel used to test the performance of the equalizers was represented by the transfer function, defined by $h=\left[\begin{array}{ccc} 0.18 & 0.85 & 0.32 \end{array}\right]$.

**Figure 11:**
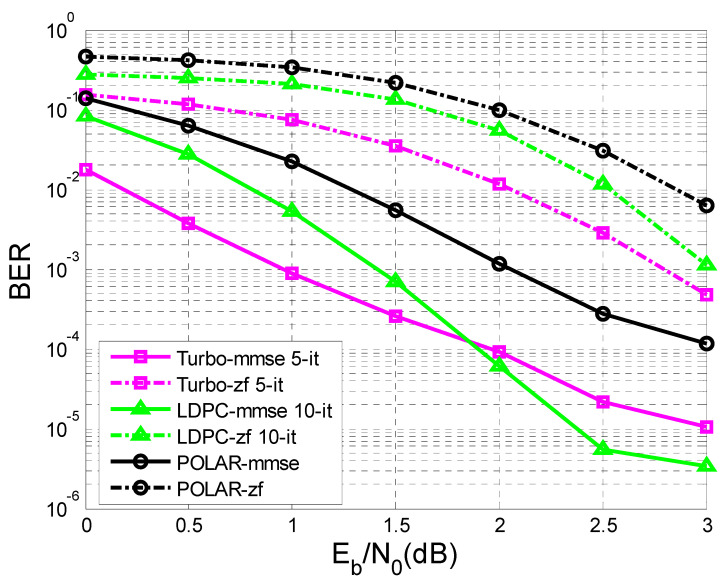
BER vs. SNR for different codes in the case of MMSE and ZF equalization in the case of perfect channel estimation (K = 342 bits and h = (0.18 0.85 0.32)).

**Figure 12:**
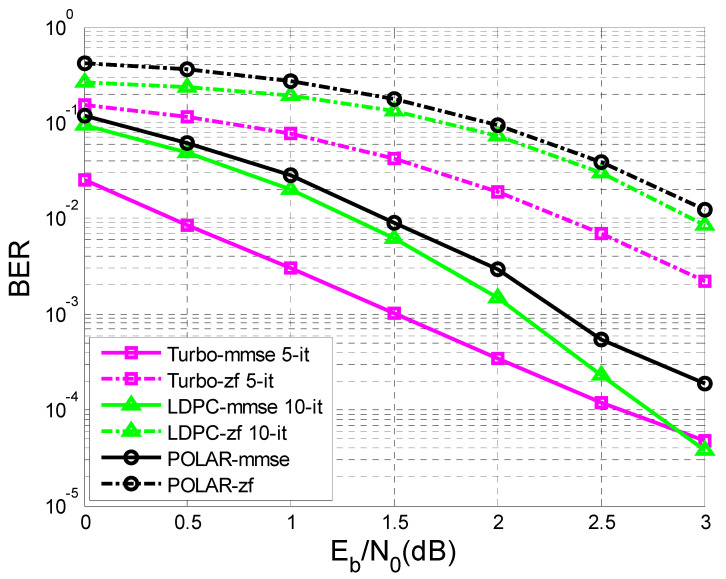
BER vs. SNR for different codes in the case of MMSE and ZF equalization in the case of perfect channel estimation (K = 170 bits and h = (0.18 0.85 0.32)).

In Figure 11 and Figure 12, the difference in the performance that the MMSE (minimum mean square error) equalization provided, in terms of BER vs. SNR, before the decoding process compared to the ZF (Zero Forcing) equalization is very obvious.

In Figure 11 the number of transmitted sequences was 10,000, with 342 bits of information each, in comparison with Figure 12, where the number of transmitted sequences was 10,000, with 170 bits of information each. 

It was observed that the performance became better as the length of the sequence of information bits became longer. For a given transfer function, the longer the sequence of information bits, the better the performance obtained for the same SNR.

In the simulation process, turbo decoding used five iterations, and LDPC decoding used 10 iterations.

In Figure 12, at a given SNR of 3 dB, if the length of the transmitted sequence is 170 bits, it is observed that the performance of the LDPC codes with 10 iterations and turbo codes with five iterations were close using MMSE equalization.

If the length of the transmitted sequence was 342 bits, as in Figure 11, similar performances using MMSE equalization were obtained for a given SNR of 2 dB, regarding the LDPC codes and turbo codes. 

Moreover, in the case of Figure 11 and Figure 12, for a given SNR of 3 dB using MMSE equalization, the LDPC codes with 10 iterations outperformed the turbo codes with five iterations. 

At the same time, in Figure 11, regarding the case of ZF equalization, a convergence of the performance of LDPC codes and turbo codes could be observed with the increase of the transmitted data sequence, but for a higher SNR than in the case of MMSE equalization.

Following Figure 12, if K = 170 bits, the BER value for LDPC-mmse 10-it for an SNR of 3dB is equal to $\frac{1}{7}\times 10^{-4}$, and the BER value for Turbo-mmse 5-it for the same SNR is $\frac{1}{6}\times 10^{-4}$. If K = 342 bits (Figure 11), the BER value for LDPC-mmse 10-it for an SNR of 3 dB is $\frac{1}{8}\times 10^{-5}$, and the BER value for Turbo-mmse 5-it for the same SNR is $10^{-5}$. Thus, an improvement of BER vs. SNR was observed, for the same SNR, as K increased. In other words, BER decreased as K increased. Furthermore, according to [47], LDPC codes were the best performing, even outperforming turbo codes.

**Figure 13:**
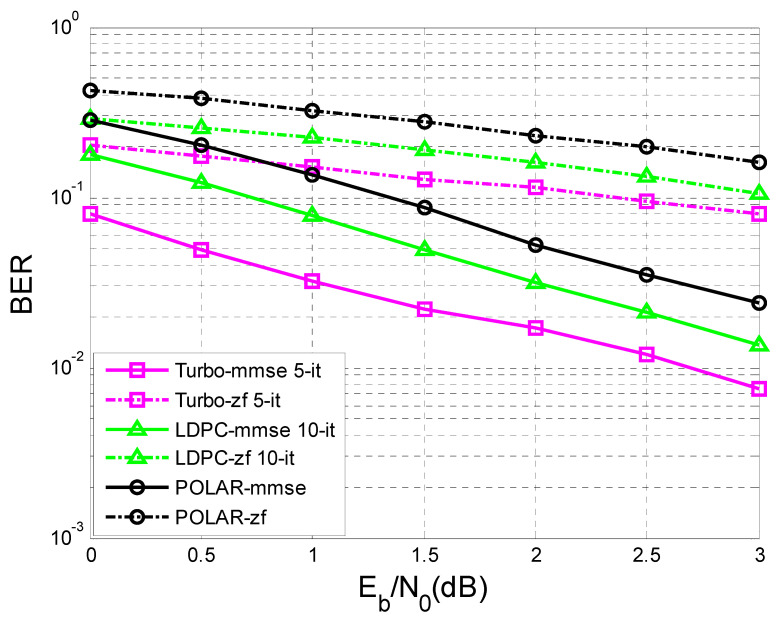
BER vs. SNR for different codes in the case of MMSE and ZF equalization applying LS estimation (K = 342 bits h = (0.18 0.85 0.32), N = 16).

**Figure 14:**
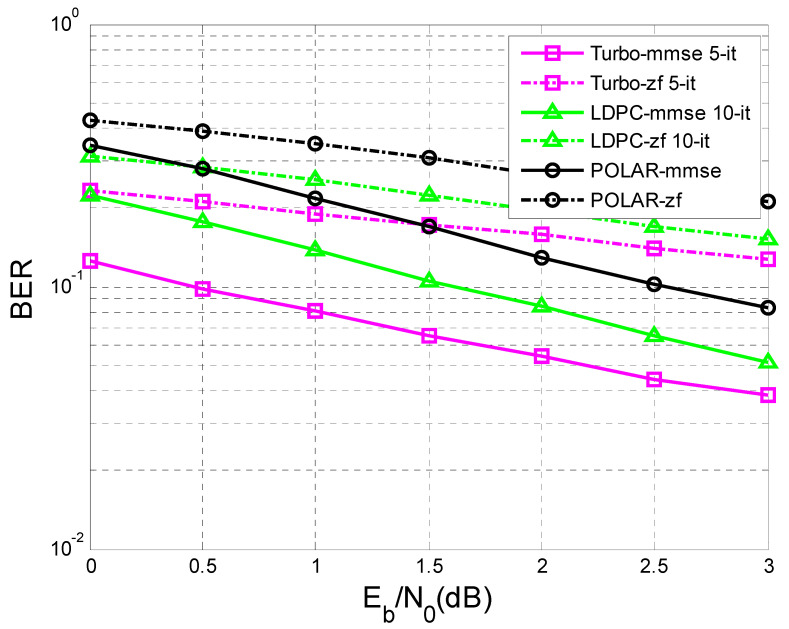
BER vs. SNR for different codes in the case of MMSE and ZF equalization applying LS estimation (K = 342 bits h = (0.18 0.85 0.32), N = 8).

Figure 13 and Figure 14 present the results obtained for the ZF and MMSE equalization in the case when the channel response to the impulse was estimated once only using the LS algorithm, based on a learning sequence with a length of N = 16 (Figure 13), respectively N = 8 (Figure 14). 

At this point, the specifications from the previous section related to Figure 7 and Figure 8, when the channel estimation was performed only one time, could be taken into account.

At the same time, the length of the data sequence transmitted was considered the same (K = 342 bits) in both cases.

Both figures show that the MMSE equalization was more efficient than the ZF equalization with respect to BER vs. SNR.

In the case of a larger training sequence (Figure 13), the bit error rate at a signal to noise ratio of 3 dB for all three types of codes analyzed (Turbo codes, LDPC codes, and Polar codes) was lower compared to the case in which the training sequence was considered N = 8 (Figure 14), both for MMSE equalization and ZF equalization. The estimation of h became better as the training sequence was lengthened.

Turbo codes had a better performance than LDPC codes, both for MMSE equalization and ZF equalization, meaning that at the same value of signal to noise ratio, turbo codes provide a lower level of BER.

**Figure 15:**
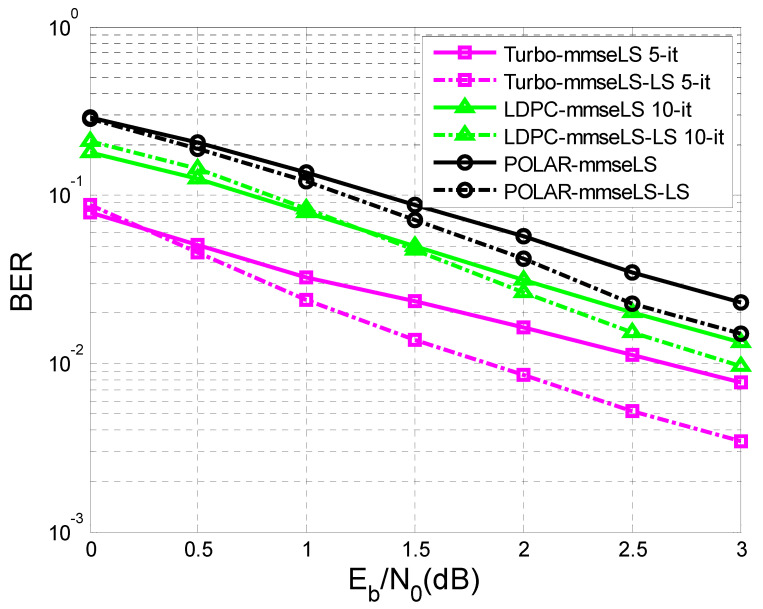
BER vs. SNR for different codes in the case of MMSE equalization applying iterative LS estimation (K = 342 bits h = (0.18 0.85 0.32), N = 16).

**Figure 16:**
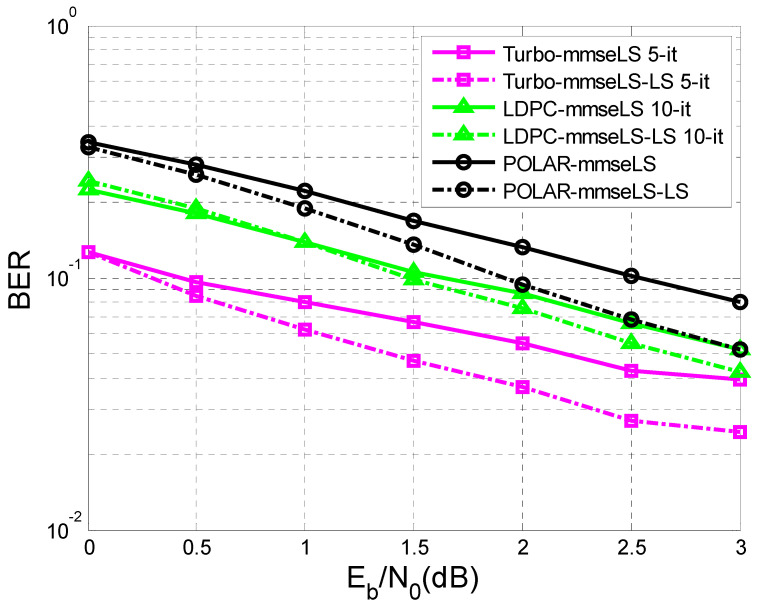
BER vs. SNR for different codes in the case of MMSE equalization applying iterative LS estimation (K = 342 bits h = (0.18 0.85 0.32), N = 8).

Finally, Figure 15 and Figure 16 illustrate the results obtained in the case of the iterative LS estimation of the channel response to the impulse h. 

Figure 15 shows the results related to the performances of turbo codes, LDPC codes, and polar codes obtained when the first estimation of h was made based on a training sequence of N = 16. 

Figure 16 presents the same elements in the condition where the length of the training sequence was N = 8. 

In both situations, the following estimates of h were achieved based on the sequence obtained at the output of the decoder ${\hat{b}}_{k}$ using the same LS algorithm. In addition, the specifications from the previous section related to Figure 9 and Figure 10, in the case where the channel estimation was performed iteratively, could be taken into consideration.

As a matter of fact, in both situations, only MMSE equalization was used, because it proved to be more efficient than ZF equalization.

It was observed that the iterative LS estimation of h offered substantial improvements in the case of all three codes than its estimation only once.

The better the initial estimation of h (Figure 15), the lower level of BER was obtained for a certain value of SNR, compared to the case presented in Figure 16, where the training sequence was smaller and involved a higher number of iterations to reach the same level of BER, as in the situation depicted in Figure 15.

This paper is limited to the performance analysis of turbo codes, LDPC codes, and polar codes on an AWGN channel in the presence of inter symbol interference. As was mentioned in the Section 1, to mitigate the negative effect of interference it is necessary to apply channel equalization. Therefore, to perform this analysis, only the BER vs. SNR curves for turbo codes, LDPC codes, and polar codes, on an AWGN channel with ISI, were drawn in Figure 11, Figure 12, Figure 13, Figure 14, Figure 15 and Figure 16.

Of course, the analysis of the performance of turbo codes, LDPC codes, and polar codes in the condition in which the impulse response of the channel was considered to be unitary (meaning the ideal situation) was carried out in the papers [19,20], where the BER vs. SNR curves for turbo codes, LDPC codes, and polar codes, using an AWGN channel without ISI, were drawn and where a much lower level of BER for a given SNR was observed.

Although, currently, OFDM is predominantly used, this fact should not impede/stop comparisons of the performances of turbo codes, LDPC codes, and polar codes using BPSK modulation.

Indeed, the BER level is higher if the equalization is performed separately, before the decoding process, such as in this paper, and not within the decoding process, such as in turbo equalization (which is not the topic of this paper).

In this paper, the transfer function was chosen arbitrarily, and of course, depending on the response of the channel to the impulse, each code can be optimized to obtain the maximum performance.

In addition, in this paper, we did not propose any very innovative ideas regarding the three codes, but we tried to test their performance in a certain arbitrarily chosen situation. We believe that these results can be useful to other researchers, considering that we have not found any works in which the three codes were compared under the same conditions, simultaneously.

## 6. Conclusions

The higher the number of iterations in the estimation process, the more accurately the channel estimation will be performed, but the number of iterations in the decoding process will be increased considerably. For example, in the case of turbo decoding, a new estimation of h is made every five iterations. In the case of LDPC codes, after ten iterations, a new estimation of h is done, which proves that iterative LS estimation is time consuming.

If $\hat{h}$ was recalculated R times, and let I be the number of iterations performed by the decoder within a decoding process, then the total number of iterations performed by the turbo decoder, for example, is (R+1)⋅I. In the simulations realized for the scheme in Figure 9, the recalculation of $\hat{h}$ was performed only once R=1, and the number of iterations performed by the turbo decoder within a decoding process was five. It follows that in this case, the minimum number of iterations performed by the turbo decoder in Figure 9 is ten.

The performance obtained with iterative estimation will never be able to exceed the performance obtained in the case of a perfect estimation of h.

In this research, equalization was performed before decoding for the three analyzed codes, but in the future, it would be interesting to research what happens if the equalization is performed within the decoding process, such as in turbo equalization.

## Data Availability

Not applicable.
